# Clinical characteristics of children hospitalized with cellulitis in Spain (2016–2022). A population-based analysis

**DOI:** 10.3389/fped.2025.1675978

**Published:** 2025-10-28

**Authors:** Isabel Belinchón-Romero, José-Manuel Ramos-Rincón

**Affiliations:** ^1^ Department of Clinical Medicine, Miguel Hernández University, Alicante, Spain; ^2^ Department of Dermatology, Dr. Balmis General University Hospital, Alicante, Spain; ^3^ Alicante Institute for Health and Biomedical Research (ISABIAL), Alicante, Spain; ^4^ Division of Infectious Disease, Dr. Balmis General University Hospital, Alicante, Spain; ^5^ Department of Internal Medicine, Dr. Balmis General University Hospital, Alicante, Spain

**Keywords:** cellulitis, children, hospitalization, Spain, skin and soft tissue infections (ssti)

## Abstract

**Aim:**

To estimate hospitalization rates, describe clinical characteristics, and assess the direct healthcare costs of cellulitis-related hospitalizations in children of different ages: 0–4 years, 5–9 years, and 10–14 years.

**Materials and methods:**

This retrospective, population-based study included patients aged 14 years or less and hospitalized for cellulitis in Spain between 2016 and 2022. Data were obtained from the Spanish Registry of Specialist Care Activities provided by the Ministry of Health.

**Results:**

A total of 15,497 cellulitis-related hospitalizations were included: 7,378 (47.6%) aged 0–4 years, 4,532 (29.2%) aged 5–9 years, and 3,587 (23.1%) aged 10–14 years. The proportion of boys (56.6%) was higher than for girls and increased with age (*p* < 0.001). The most common anatomical site was the extremities (excluding fingers and toes), comprising 40.9% of cases, with frequency increasing with age (*p* < 0.001). Facial cellulitis was the second most frequent site (37.4%), but its proportion decreased with age (*p* < 0.001). The main predisposing factors for infection were trauma and open wounds (13.5%), especially in older children (*p* < 0.001), along with atopic dermatitis (3.1%). Only 2.8% of children required intensive care unit (ICU) admission, with the highest rates in the youngest group and the lowest in the 5–9-year-old group. In-hospital mortality was consistently low (0.1%) across age groups. The total direct cost of hospitalizations was estimated at €54.7 million: €28.2 million in 0–4-year-olds, €13.7 million in 5–9-year-olds, and €12.8 million in 10–14-year-olds.

**Conclusions:**

The clinical profile of cellulitis varies by age group. While ICU admission and in-hospital mortality were rare, the economic burden remains substantial, particularly among younger children.

## 1 Introduction

Cellulitis is a common bacterial skin infection, and the associated hospital admissions impose significant costs worldwide (1). Although it can affect individuals of all ages, in children, skin and soft tissue infections such as cellulitis, folliculitis, and impetigo represent a substantial burden on healthcare systems (2, 3).

The most frequent causative agents of cellulitis and erysipelas are *Streptococcus* spp. and *Staphylococcus aureus* (3). Known risk factors include prior episodes of cellulitis, open wounds or ulcers, tinea pedis, chronic lymphedema, venous insufficiency, skin conditions causing excoriation, and obesity (4, 5). While the epidemiology of cellulitis is well described in adults (1, 4, 5), data on pediatric hospitalizations are more limited (2, 6), except those unrelated to orbital or periorbital cellulitis (7, 8). Most studies in children focus on emergency care rather than inpatient admissions (6).

Since childhood is a stage characterized by unique exposures and risk factors for skin infections, and because the literature on pediatric hospitalizations for cellulitis (beyond orbital/periorbital cases) is limited, it is essential to characterize the occurrence and clinical impact of the disease in individuals younger than 15 years (2, 6–8). Thus, this study aimed to estimate hospitalization rates, describe the clinical characteristics, and assess the economic impact of these admissions by age group.

## 2 Methods

### 2.1 Study design

This population-based, cross-sectional study included children aged under 15 years who were hospitalized with cellulitis in any public and private hospital in Spain from 1 March 2016–31 December 2022. Data were obtained from the Spanish Registry of Specialist Care Activities, maintained by the Spanish Ministry of Health. This registry includes the minimum basic dataset for hospital discharges, which contains demographic, administrative, and clinical data, including diagnoses and procedures performed for all hospitalizations in Spain. All diagnoses and procedures were coded according to the International Classification of Diseases, 10th Revision (ICD-10) (9). The Ministry of Health periodically audits the registry to ensure data quality and accuracy.

Data collected included discharge dates and information on cellulitis hospitalizations coded using the ICD-10: L03.x (cellulitis and acute lymphangitis). This code encompasses cellulitis and acute lymphangitis while excluding: K61.x (cellulitis of anal and rectal regions), H60.1 (cellulitis of external auditory canal), N76.4 (cellulitis of external genital organs in female), N48.2, N49.x (cellulitis of external genital organs in male), H00.0 (cellulitis of eyelid), H04.3 (cellulitis of eyelid), K12.2 (cellulitis of mouth), J34.0 (cellulitis of nose), L98.3 (eosinophilic cellulitis or Wells syndrome), L98.2 (febrile neutrophilic dermatosis or Sweet syndrome), L98.2 (subacute and chronic lymphangitis). The code L03.x includes the following specific categories: L03.0 (cellulitis of finger and toe (infection of nail, onychia, paronychia, perionychia), L03.1 [cellulitis of other parts of limb (axillae, hip, shoulder)], L03.2 (cellulitis of face), L03.3 [cellulitis of trunk (abdominal wall, back, chest wall, groin, perineum, umbilicus)], L03.8 [cellulitis of other sites (head, scalp) except face], and L03.9 (cellulitis, unspecified) (Table 1).

**Table 1:** International classification of diseases, 10th edition codes used in the study.

Diagnosis	Code
Cellulitis^a^	L03.x
Cellulitis of finger and toe	L03.0
Cellulitis of other parts of limb	L03.1
Cellulitis of face	L03.2
Cellulitis of trunk	L03.3
Cellulitis of other sites	L03.8
Cellulitis, unspecified	L03.9
Comorbidities	
Diabetes mellitus type I	E10.x;
Diabetes mellitus type II	E11.x; E13.x
Neoplasms	C01.x to C80.x
Obesity	E66.x; Z68.3; Z68.4
Leukemia	C92; C93; C94; C95; C96
Lymphoma	C81 to C91
Transplantation	Z94.x
Local predisposition	
Trauma and open wounds	S00.x to S99.x
Abrasions	T14.x
Atopic dermatitis	L20.x
Psoriasis	L40.x
Immunodeficiencies	
HIV	B20.x
Immunoglobulin deficiencies	B80.x
Combined immunodeficiencies	B81.x
Other well-defined syndromes of immunodeficiency	B82.x
Common variable immunodeficiency	B83.x
Other immunodeficiencies	B84.x
Disorders of phagocytic function	D70.x
Complications	
Sepsis	A40.x; A41.x
Severe sepsis	R65.2x
Acute kidney injury	N17.x
Microbiology	
*Escherichia coli*	
* E. coli* sepsis	A41.51
* E. coli* as the cause of diseases classified elsewhere	B96.2x
*Streptococcus* spp.	
*Streptococcal* sepsis (sepsis caused by Streptococcus)	A40.x
*Streptococcus*, group A, as the cause of diseases classified elsewhere	B95.0x
*Streptococcus*, group B, as the cause of diseases classified elsewhere	B95.1x
Other *streptococcus* as the cause of diseases classified elsewhere	B95.4x
Unspecified s*treptococcu*s as the cause of diseases classified elsewhere	B95.5x
*Staphylococcus* spp.	
Sepsis due to *Staphylococcus aureus*	A41.0x
Sepsis due to another specified *staphylococcus*	A41.1x
Sepsis due to unspecified *staphylococcus*	A41.2x
* Staphylococcus aureus* as the cause of diseases classified elsewhere	B95.6x
Other *staphylococcus* as the cause of diseases classified elsewhere	B95.7
Unspecified *staphylococcus* as the cause of diseases classified elsewhere	B95.8
*Pseudomonas* spp.	
Sepsis due to *Pseudomonas* spp.	A41.52
Pseudomonas as the cause of diseases classified elsewhere	B96.5

**L03.0 Cellulitis of finger and toe** (infection of nail**,** Onychia**,** Paronychia**,** Perionychia.

**L03.1 Cellulitis of other parts of limb** (axille, hip**,** shoulder).

**L03.2 Cellulitis of face.**

**L03.3 Cellulitis of trunk** (abdominal wall), back [any part], chest wall, groin, perineum, umbilicus. ***Excl.:*** omphalitis of newborn (P38).

**L03.8 Cellulitis of other sites:** head [any part, except face], scalp.

**L03.9 Cellulitis, unspecified.**

^a^ **L03 cellulitis: *Incl.:*** acute lymphangitis ***Excl.:*** cellulitis of: anal and rectal regions (K61.-) external auditory canal (H60.1), external genital organs: (female (N76.4), male (N48.2, N49.-), eyelid (H00.0), lacrimal apparatus (H04.3), mouth (K12.2, nose (J34.0); eosinophilic cellulitis [Wells] (L98.3); febrile neutrophilic dermatosis [Sweet] (L98.2); lymphangitis (chronic)(subacute) (I89.1).

### 2.2 Variables

We analyzed: (a) demographic variables: age group (0–4, 5–9, and 10–14 years) and sex; (b) clinical characteristics: type and location of cellulitis (by ICD-10 code), and comorbidities [e.g., lymphoma, malignancies, obesity, diabetes, HIV, and primary immunodeficiencies (immunoglobulin deficiencies, combined immunodeficiencies, other well-defined syndromes of immunodeficiency and disorders of phagocytic function)]; (c) microbiological data: infections due to *Staphylococcus* spp., *Streptococcus* spp., *Escherichia coli*, and *Pseudomonas* spp.; (d) severe clinical outcomes, including sepsis, severe sepsis, acute kidney injury, and intensive care unit (ICU) admission; and (e) hospital outcomes: length of stay, type of discharge (home or deceased), and cost of hospitalization. Table 1 includes the full list of ICD-10 codes for diagnoses, comorbidities (including immunodeficiencies), and microbiological data.

Sepsis was identified using ICD-10 codes A40.x (streptococcal sepsis) and A41.x (other bacterial sepsis, including *S. aureus*, *Haemophilus influenzae*, anaerobes, and other Gram-negative organisms). Severe sepsis was defined using R65.2, indicating infection with acute organ dysfunction. The underlying infection was always coded first.

Microbiological pathogen identification was based on ICD-10 codes registered in the national hospital discharge database. We included the following categories: *E. coli* (A41.51, B96.2x), *Streptococcus* spp. (A40.x, B95.0x, B95.1x, B95.4x, B95.5x), *Staphylococcus* spp. (A41.0x, A41.1x, A41.2x, B95.6x, B95.7, B95.8), and *Pseudomonas* spp. (A41.52, B96.5). In this dataset, a coded diagnosis of microbial infection was interpreted as culture-confirmed in the clinical setting (blood or another sterile site). However, the database does not provide access to individual microbiological reports, so we could not verify culture source or method of isolation (Table 1).

Hospitalization costs were estimated using the diagnosis-related group (DRG) system. Cost data were derived from clinical-administrative records in the hospital discharge database and hospital accounting databases, audited annually by the Ministry of Health (10).

### 2.3 Data analysis

The hospitalization rate per 1,000 admissions was calculated as the number of hospitalizations for cellulitis per 1,000 all-cause pediatric hospital admissions per year, using data from the hospital discharge database (11). In addition, the annual hospitalization rate for cellulitis was calculated as the number of hospitalizations for cellulitis per 100,000 children in the population, using age-specific annual population estimates from the Spanish National Institute of Statistics (12).

Categorical variables were reported as absolute counts and percentages. Continuous variables were summarized as medians with interquartile ranges (IQRs). Normality was assessed using the Kolmogorov–Smirnov test. Comparisons across groups were performed using the chi-squared test for categorical variables and the Kruskal–Wallis test for continuous variables. *P* values under 0.05 were considered statistically significant. Analyses were conducted using IBM SPSS for Windows, version 25.0 (IBM Corp., Armonk, NY, USA).

### 2.4 Ethical aspects

The Spanish Ministry of Health provided the anonymized dataset after removing all personal identifiers. According to Spanish legislation, informed consent is not required for studies using anonymized administrative databases. The Ethics and Research Integrity Committee of Miguel Hernández University approved the research protocol (Ref. AUT.DMC.JMRR.241103), which adhered to the ethical principles of the revised Declaration of Helsinki (2013).

## 3 Results

### 3.1 Annual trends in case numbers and hospitalization rates by age group

During the study period, there were a total of 1.88 million hospital admissions for children under 15 years of age in Spain (≈1.2 million aged 0–4 years, 339,000 aged 5–9 years, and 343,000 aged 10–14 years). Of these, 15,497 were due to cellulitis: 7,378 (47.6%) in 0–4-year-olds, 4,532 (29.2%) in 5–9 years group, and 3,587 (23.1%) in the 10–14 years group.

**Figure 1:**
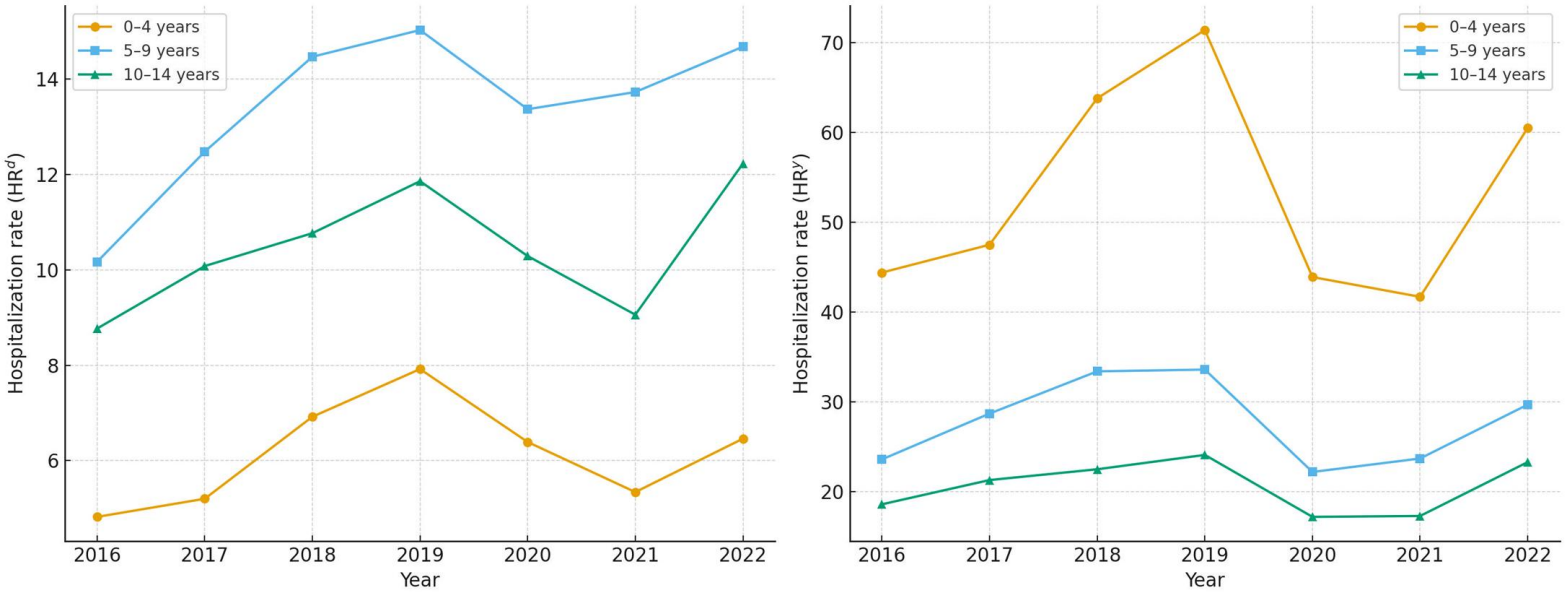
Annual hospitalization rates for cellulitis in children by age group, Spain, 2016–2022. **(A)** Hospitalization rate per admissions (HR^ad^): number of cellulitis hospitalizations per 1,000 all-cause pediatric hospital admissions. **(B)** Yearly hospitalization rate (HR^y^): number of cellulitis hospitalizations per 100,000 children in the population. Rates were calculated using the Spanish national hospital discharge database (population estimates from the National institute of statistics).

The annual number of cellulitis-related hospitalizations increased from 1971 cases in 2016 to a peak of 2813 in 2019, then declined to 1,737 cases in 2021 during the COVID-19 pandemic, before rising again to 2,339 in 2022. This temporal pattern was consistent across all age groups, with a marked decline during the pandemic years (Table 2; Figure 1).

**Table 2:** Episodes of cellulitis hospitalization in children, hospitalization rates per 1,000 all-cause admissions and per 100,000 inhabitants by age group in Spain.

Year	Total			0–4 years			5–9 years			10–14 years		
	*N*	HR^ad^	HR^y^	*N*	HR^ad^	HR^y^	*N*	HR^ad^	HR^y^	*N*	HR^ad^	HR^y^
2016	1,971	6.48	28.3	950	4.82	44.4	578	10.17	23.6	443	8.77	18.6
2017	2,204	7.40	31.8	994	5.20	47.5	692	12.48	28.7	518	10.08	21.3
2018	2,653	8.99	38.5	1,309	6.92	63.8	785	14.47	33.4	559	10.77	22.5
2019	2,813	9.94	41.2	1,426	7.92	71.4	779	15.03	33.6	608	11.86	24.1
2020	1,780	8.42	26.5	840	6.39	43.9	505	13.37	22.2	435	10.29	17.2
2021	1,737	7.52	26.3	770	5.34	41.7	532	13.73	23.7	435	9.06	17.3
2022	2,339	8.93	35.7	1,089	6.46	60.5	661	14.68	29.7	589	12.23	23.3
*Mean*	*2,213*	*8*.*20*	*32*.*1*	*1,054*	*6*.*10*	*52*.*3*	*647*	*13*.*3*	*27*.*5*	*512*	*10*.*4*	*20*.*4*

HR*^ad^*: Number of hospitalizations for cellulitis per 1,000 all-cause admissions per year.

HR*^y^*: Number of hospitalizations for cellulitis per 100,000 inhabitants per year.

The mean annual hospitalization rate was 8.2 per 1,000 total admissions and 32.1 per 100,000 inhabitants. When stratified by age, the hospitalization rate per 1,000 admissions was lower in the 0–4 age group (6.1) compared to the 5–9 (13.3) and 10–14 age groups (10.4). However, the hospitalization rate per 100,000 population was higher in the youngest group (52.3 compared to 27.5 in children aged 5–9 years and 20.4 in those aged 10–14 years) (Table 2; Figure 1).

### 3.2 Clinical characteristics by age group

Table 3 presents the demographic, clinical, and outcomes by age group. Overall, there was a higher proportion of boys (56.6%) compared to girls (43.4%), and the male predominance increased with age (*p* < 0.001).

**Table 3:** General characteristics and direct costs (€) of cellulitis hospitalizations in children in Spain, by age group.

Variables	Total	0–4 years	5–9 years	10–14 years	*P* value^b^
	(*N* = 15,497)	(*N* = 7,378)	(*N* = 4,532)	(*N* = 3,587)	
	*n* (%)^a^	*n* (%)^a^	*n* (%)^a^	*n* (%)^a^	
Sex					
Boy	8,768 (56.6)	4,077 (55.3)	2,573 (58.8)	2,118 (59.0)	**0** **.** **001**
Girld	6,727 (43.4)	3,301 (44.7)	1,957 (43.2)	1,469 (41.0)	
Location of cellulitis^c^					
Other parts of limb [L03.1]	6,331 (40.9)	2,560 (34.7)	1,987 (43.8)	1,784 (49.7)	**<0** **.** **001**
Face [L03.2]	5,802 (37.4)	2,977 (40.3)	1,758 (38.8)	1,067 (29.7)	**<0** **.** **001**
Finger and toe [L03.0]	1,598 (10.3)	870 (11.8)	321 (7.1)	407 (11.3)	**<0** **.** **001**
Trunk [L03.3]	1,300 (8.4)	772 (9.8)	349 (7.7)	229 (6.4)	**<0** **.** **001**
Unspecified [L03.9]	367 (2.4)	197 (2.7)	101 (2.2)	69 (1.9)	**0** **.** **042**
Other sites [L03.8]	312 (2.0)	159 (2.2)	82 (1.8)	71 (2.0)	0.42
Microbiology (ICD-10)					
* Staphylococcus* spp.	1,791 (11.6)	912 (12.4)	432 (9.5)	447 (12.5)	**<0** **.** **001**
* Streptococcus* spp.	418 (2.7)	208 (2.8)	108 (2.4)	102 (2.8)	0.30
* Escherichia coli*	112 (0.7)	60 (0.8)	25 (0.6)	27 (0.8)	0.24
* Pseudomonas* spp.	155 (1.0)	69 (0.9)	30 (0.7)	56 (1.8)	**<0** **.** **001**
Comorbidities					
Lymphoma	258 (1.7)	97 (1.3)	77 (1.7)	84 (2.3)	**<0** **.** **001**
Neoplasia	164 (1.1)	63 (0.9)	45 (1.0)	56 (1.6)	**0** **.** **003**
Obesity	144 (0.9)	13 (0.2)	31 (0.7)	100 (2.8)	**<0** **.** **001**
Leukemia	93 (0.4)	28 (0.4)	9 (0.2)	26 (0.7)	**0** **.** **001**
Transplantation	60 (0.4)	21 (0.3)	15 (0.3)	24 (0.7)	**0** **.** **013**
Diabetes mellitus type 1	47 (0.3)	7 (0.1)	11 (0.2)	29 (0.8)	**<0** **.** **001**
Diabetes mellitus type 2	3 (0.0)	1 (0.0)	1 (0.0)	1 (0.0)	0.87
Local predisposition					
Trauma and open wounds	2,096 (13.5)	875 (11.9)	679 (15.0)	542 (15.1)	<0.001
Atopic dermatitis	473 (3.1)	225 (3.0)	150 (3.3)	98 (2.7)	0.32
Psoriasis	21 (0.19	6 (0.6)	7 (0.2)	8 (0.8)	0.15
Abrasions	20 (0.1)	6 (0.1)	8 (0.2)	6 (0.2)	0.28
Immunodeficiencies					
Immunoglobulin deficiencies	30 (0.2)	15 (0.29	9 (0.2)	6 (0.2)	0.91
Other immunodeficiencies	19 (0.1)	7 (0.19	6 (0.1)	6 (0.2)	0.58
Disorders of phagocytic function	7 (0.0)	2 (0.0)	3 (0.19	2 (0.1)	0.58
Other well-defined syndromes of immunodeficiency	7 (0.0)	0 (0.0)	5 (0.1)	2 (0.1)	0.02
Combined immunodeficiencies	5 (0.0)	2 (0.0)	2 (0.0)	1 (0.0)	0.89
Common variable immunodeficiency	2 (0.0)	0 (0.0)	0 (0.0)	2 (0.1)	0.05
HIV	1 (0.0)	0 (0.0)	0 (0.0)	0 (0.0)	0.19
Clinical complications					
Sepsis	168 (1.1)	107 (1.5)	25 (0.6)	36 (1.0)	**<0** **.** **001**
Severe sepsis	60 (0.4)	31 (0.4)	13 (0.3)	16 (0.4)	0.41
Acute kidney failure	45 (0.3)	18 (0.2)	9 (0.2)	18 (0.5)	0.35
Outcome					
Length of stay, median (IQR)	4 (2–6)	4 (2–6)	3 (2–5)	4 (3–6)	**<0** **.** **001**
ICU admission^d^	431 (2.8)	240 (3.3)	19 (1.8)	112 (3.2)	**<0** **.** **001**
Days in ICU, median (IQR)^e^	3 (0–10)	4 (0–16)	2 (0–8)	2 (0–5.5)	**<0** **.** **001**
In-hospital mortality	17 (0.1)	11 (0.1)	3 (0.1)	3 (0.1)	0.36
Direct costs (€)					
Absolute cost	54,718,584	28,217,678	13,741,328	12,759,578	**<0** **.** **001**
Mean cost per admission (SD)	3,531 (5,252)	3,824 (6,405)	3,032 (3,216)	3,557 (4,606)	**<0** **.** **001**
Median cost per admission (IQR)	2,439 (2,312–3,237)	2,439 (2,312–3,061)	2,415 (2,312–2,647)	2,439 (2,312–5,092)	**<0** **.** **001**

COPD, chronic obstructive pulmonary disease; ICD-10, international classification of diseases, 10th Revision; ICU, intensive care unit, IQR, interquartile range, SD, standard deviation.

^a^ Unless otherwise indicated.

^b^ In bold, *p* value < 0.05. Analyses were conducted using the chi-squared test (3  ×  2) for categorical variables and the Kruskal–Wallis test for continuous variables.

^c^ ICD cod. The sum of all locations may exceed the total number of patients, as some patients may have been recorded in more than one location.

^d^ Missing value *n* = 131: 0–4 years, *n* = 53; 5–9 years, *n* = 42; 10–14 years, *n* = 36.

^e^ Available *n* = 431.

The most frequent anatomical location of cellulitis was the limbs (excluding fingers and toes), accounting for 40.9% of cases, with this proportion increasing with age (*p* < 0.001). The second most frequent site was the face (37.4%), which showed the opposite trend (*p* < 0.001). Less frequent sites included the fingers and toes (10.3%) and the trunk (8.4%), both of which also decreased with age (*p* < 0.001).

Comorbidities were rare but most prevalent in older children. The most common were lymphoma (1.7%), neoplasms (1.1%), and obesity (0.9%). Immunodeficiencies were also rare, with immunoglobulin deficiencies being the most common (0.2%). Local predisposing factors for infection were more frequent, particularly trauma and open wounds (13.5%), especially in older children (*p* < 0.001), followed by atopic dermatitis (3.1%).

The overall prevalence of sepsis was 1.1%. Staphylococcal infection was the most frequently identified pathogen (11.6%), though it was less common in the 5–9-year age group. Streptococcal infections were recorded in 2.7% of cases.

The median length of hospital stay was 4 days [interquartile range (IQR) 2–6], shortest in the 5–9 years group, followed by the 10–14 group, and slightly longer in the 0–4 years group. Only 2.8% of patients required admission to the ICU, with the lowest rate in the 5–9 years group and the highest in the 0–4 years group (*p* < 0.001). ICU stays were also longer in younger children (*p* < 0.001). In-hospital mortality was low (0.1%) and did not differ significantly across age groups.

### 3.3 Direct healthcare costs of cellulitis hospitalizations by age group

The total direct cost of the 15,497 cellulitis-related hospitalizations in children over the seven-year period was estimated at €54.7 million, distributed as follows by age group: €28.2 million for the 0–4 years group, €13.7 million for the 5–9 years group, and €12.8 million for the 10–14 years group (Table 3). The median cost per admission was €2,439, with the highest costs observed in the 0–4 years group, followed by the 10–14 and 5–9 years groups.

## 4 Discussion

This study provides a comprehensive analysis of the epidemiological and clinical characteristics of pediatric hospitalizations for cellulitis in Spain, stratified by age group, based on data from a national administrative database. We observed an increasing trend in hospitalizations up to 2019, followed by a marked decline during the COVID-19 pandemic, a pattern consistent with reduced admissions for other infectious diseases during that period.

Interestingly, while children aged 0–4 years had lower hospitalization rates per 1,000 hospital admissions compared to older age groups, they exhibited the highest rates per 100,000 child-year. This reflects the higher population denominator in this age group and suggests that although cellulitis is an uncommon cause of admission in young children (13, 14), it remains a significant public health concern due to the size of the population.

The drop in hospitalizations during the COVID-19 pandemic likely reflects the broader reduction in the circulation of infectious diseases due to public health measures, as seen in other studies (15, 16).

Regarding the anatomical distribution of cellulitis, the limbs—excluding fingers and toes—were the most frequent site of infection, particularly among older children. In contrast, facial cellulitis was more prevalent in the 0–4 years age group. These findings are consistent with previous studies reporting that orbital and periorbital cellulitis is more common in younger children, often resulting from the anatomical proximity of the ethmoid sinuses and their early development (7), as well as trauma or hematogenous spread (8).

The most frequently identified pathogens were *Staphylococcus* spp., followed by *Streptococcus* spp. In particular, *Staphylococcus aureus* and *Streptococcus pyogenes* are generally considered the primary causative agents of cellulitis (6, 17–19). Differences in prevalence reported across studies may reflect variations in the type of cellulitis (e.g., orbital vs. limb), care setting, age distribution, or geographic factors (19). For example, since September 2022 there has been an increase in group A *Streptococcus* infection in Europe, including severe skin and soft tissue infections (20); however, our study covered cases only until December 2022, which may explain the lower prevalence observed. Other microorganisms occasionally responsible for cellulitis include *E. coli* and *Pseudomonas* spp., typically in immunocompromised patients or those with chronic skin ulcers (21, 22). In our study, these pathogens accounted for less than 1% of cellulitis cases.

Predisposing conditions play a central role in the development of cellulitis in children. Previous studies have highlighted that skin barrier disruption facilitates bacterial entry and increases the risk of soft tissue infection; such disruptions can stem from minor trauma, insect bites, abrasions, or dermatological conditions such as atopic dermatitis or psoriasis (2, 23). Other chronic comorbidities, such as oncohematological malignancies (24), obesity (25), and primary immunodeficiency (26) have also been described as risk factors, but they are much less frequent in the pediatric population compared with adults (1). Predisposing factors are not always clearly identified in all cases (27).

In our study, consistent with this evidence, the most common predisposing factors were trauma and open wounds, present in 13.5% of cases, and were particularly frequent in older children. Atopic dermatitis was also identified as a contributing factor, albeit less commonly (3.1%). In contrast, systemic comorbidities such as immunodeficiencies or malignancies were rare. These findings support the notion that, in children, cellulitis is predominantly triggered by local disruption of the skin barrier.

ICU admissions were rare, occurring more often in the youngest children as seen in other studies (28), and the overall in-hospital mortality rate was low (0.1%), lower than that reported in adults with cellulitis (29). This suggests that severe outcomes are uncommon in pediatric patients, even when hospitalized.

Regarding the economic burden, the total estimated cost of hospitalizations over the study period was €54.7 million. Although substantial, these costs are lower than those reported for adults in Spain (29). Pediatric cellulitis remains a relevant contributor to healthcare expenditures and resource utilization (30).

A major strength of this study is its national scope and age-stratified analysis, offering a robust population-level perspective on pediatric cellulitis hospitalizations. The use of administrative data enabled the inclusion of a large sample size, allowing for reliable estimates of hospitalization rates, clinical patterns, and associated costs (31).

Nevertheless, several limitations should be acknowledged. First, administrative databases capture hospital discharges, with each admission (including readmissions) counted as a separate episode, which may lead to overestimation. Second, clinical detail is limited to discharge diagnoses, and variability in coding practices may introduce inaccuracies or inconsistencies. Third, pathogen identification relies on coded diagnoses, which are assumed to reflect positive microbiological cultures, although culture data and specimen sites were not available. Fourth, laboratory test results (e.g., hematology, biochemistry, inflammatory markers) are not included in the administrative database. As a result, we could not evaluate analytical profiles or compare them across age groups. Similarly, the database does not contain information on therapeutic interventions or imaging studies, which precluded analysis of treatment strategies and diagnostic procedures.

This study highlights an upward trend in pediatric cellulitis hospitalizations until 2019, followed by a decline during the COVID-19 pandemic. Older children most commonly had infections in the limbs, and younger children on their face. ICU admissions and mortality were rare, emphasizing the generally favorable prognosis in this population. However, the healthcare costs are not negligible, and these findings reinforce the importance of continued surveillance and suggest that administrative health databases are a valuable tool for understanding trends in pediatric infectious diseases.

## Data Availability

The raw data supporting the conclusions of this article will be made available by the authors, without undue reservation.

## References

[1] Bystritsky RJ. Cellulitis. Infect Dis Clin North Am. (2021) 35(1):49–60. 10.1016/j.idc.2020.10.002 33494874

[2] Galli L Venturini E Bassi A Gattinara GC Chiappini E Defilippi C Common community-acquired bacterial skin and soft-tissue infections in children: an intersociety consensus on impetigo, abscess, and cellulitis treatment. Clin Ther. (2019) 41(3):532–551.e17. 10.1016/j.clinthera.2019.01.010 30777258

[3] Zha M Usatine R. Common skin conditions in children and adolescents: bacterial infections. FP Essent. (2024) 541:14–9. PMID: 38896826. 38896826

[4] Quirke M Ayoub F McCabe A Boland F Smith B O’Sullivan R Risk factors for nonpurulent leg cellulitis: a systematic review and meta-analysis. Br J Dermatol. (2017) 177(2):382–94. 10.1111/bjd.15186 27864837

[5] Ching CC Johar A. Clinical characteristics of patients with lower limb cellulitis and antibiotic usage in hospital Kuala Lumpur: a 7-year retrospective study. Int J Dermatol. (2016) 55(1):30–5. 10.1111/ijd.12850 26275796

[6] Shriner A Wilkie L. Pediatric cellulitis: a red-hot concern. Pediatr Ann. (2017) 46(7):e265–9. 10.3928/19382359-20170620-01 28697269

[7] Yadalla D Jayagayathri R Padmanaban K Ramasamy R Rammohan R Nisar SP Bacterial orbital cellulitis—a review. Indian J Ophthalmol. (2023) 71(7):2687–93. 10.4103/IJO.IJO_3283_22 37417106 PMC10491050

[8] Anosike BI Ganapathy V Nakamura MM. Epidemiology and management of orbital cellulitis in children. J Pediatric Infect Dis Soc. (2022) 11(5):214–20. 10.1093/jpids/piac006 35438766 PMC9155619

[9] Diccionario Panhispánico del Español Jurídico. Real decreto 69/2015, de 6 de febrero, por el que se regula el registro de actividad de atención sanitaria especializada. Boletín Oficial del Estado. (2025) 1235:10790–890. Available online at: https://www.sanidad.gob.es/estadEstudios/estadisticas/docs/BOE_RD_69_2015_RAE_CMBD.pdf (Accessed June 23, 2025).

[10] Agencia de Calidad del Sistema Nacional de Salud. Instituto de Información Sanitaria. Ministerio de Sanidad y Consumo. Metodología de Análisis de la hospitalización en el Sistema Nacional de Salud. Modelo de Indicadores Basado en el Registro de Altas (CMBD) Documento Base. Available online at: https://www.mscbs.gob.es/estadEstudios/estadisticas/docs/metod_modelo_cmbd_pub.pdf (Accessed June 23, 2025).

[11] RAE-CMBD. Serie histórica. Información general (SNS). Resumen por Comunidad Autónoma—Tipo de Hospital—Servicios. Available in: Available online at: https://pestadistico.inteligenciadegestion.sanidad.gob.es/publicoSNS/C/rae-cmbd/serie-historica/informacion-general-sns/resumen-por-comunidad-autonoma-tipo-de-hospital-servicios (Accessed June 23, 2025).

[12] INEbase. Cifras de Poblacón y Censos Demográficos. Estadística Continua de la Población_Resultados Definitivos. Resident Population by Date, Sex and Age (from 1971), Available online at: https://www.ine.es/jaxiT3/Tabla.htm?t=56934 (Accessed June 23, 2025).

[13] Abdalla T Hendrickx D Fathima P Walker R Blyth CC Carapetis JR Hospital admissions for skin infections among Western Australian children and adolescents from 1996 to 2012. PLoS One. (2017) 12(11):e0188803. 10.1371/journal.pone.0188803 29190667 PMC5708667

[14] Gubbay JB McIntyre PB Gilmour RE. Cellulitis in childhood invasive pneumococcal disease: a population-based study. J Paediatr Child Health. (2006) 42(6):354–8. 10.1111/j.1440-1754.2006.00872.x 16737477

[15] Ramos-Rincón JM Pinargote-Celorio H González-de-la-Aleja P Sánchez-Payá J Reus S Rodríguez-Díaz JC Impact of influenza related hospitalization in Spain: characteristics and risk factor of mortality during five influenza seasons (2016 to 2021). Front Public Health. (2024) 12:1360372. 10.3389/fpubh.2024.1360372 38628848 PMC11018950

[16] Foley DA Minney-Smith CA Tjea A Nicol MP Levy A Moore HC The changing detection rate of respiratory syncytial virus in adults in western Australia between 2017 and 2023. Viruses. (2024) 16(5):656. 10.3390/v16050656 38793538 PMC11125702

[17] Glennon CM El Saleeby C Kroshinsky D. Cellulitis in pediatric patients: recognition and management in the era of evolving resistance. Am J Clin Dermatol. (2025) 26(4):537–53. 10.1007/s40257-025-00936-w 40259138

[18] Chehab L Doody DR Esbenshade AJ Guilcher GMT Dvorak CC Fisher BT A population-based study of the long-term risk of infections associated with hospitalization in childhood cancer survivors. J Clin Oncol. (2023) 41(2):364–72. 10.1200/JCO.22.00230 35878085 PMC9839247

[19] Martinón-Torres F Salas A Rivero-Calle I Cebey-López M Pardo-Seco J Herberg JA Life-threatening infections in children in Europe (the EUCLIDS project): a prospective cohort study. Lancet Child Adolesc Health. (2018) 2(6):404–14. 10.1016/S2352-4642(18)30113-5 30169282

[20] Colomina MS Flamant A Le Balle G Cohen JF Berthomieu L Leteurtre S Severe group A streptococcus infection among children, France, 2022–2024. Emerg Infect Dis. (2025) 31(9):1698–707. 10.3201/eid3109.250245 40866794 PMC12407216

[21] Abdipour Mehrian SR Noushadi F Pourasghar Y Farkarian A Meftah E Homayounifar F Prevalence, microbiology, and antimicrobial susceptibility profile of bacterial skin and soft tissue infections in pediatric patients with malignancies at a referral teaching hospital in Shiraz, Iran. BMC Infect Dis. (2025) 25(1):707. 10.1186/s12879-025-11059-2 40380338 PMC12083172

[22] Aman BS Taj MK Taj I Azam S Khan S Rashid R. The bacterial profile and antibiotic susceptibility in skin and soft tissue infections at a tertiary care hospital of Quetta, Pakistan. J Pak Med Assoc. (2024) 74(7):1249–53. 10.47391/JPMA.10261 39028049

[23] Nicolini G Frasca Polara V Calvi M García-Lorenzo M Massese M Milani GP When infection hurts: golden rules for managing pediatric skin and soft tissue infections. Ital J Pediatr. (2025) 51(1):194. 10.1186/s13052-025-01994-w 40528194 PMC12175349

[24] Hernández Orozco H Lucas Resendiz E Castañeda JL De Colsa A Ramirez Mayans J Johnson KM Surveillance of healthcare associated infections in pediatric cancer patients between 2004 and 2009 in a public pediatric hospital in Mexico city, Mexico. J Pediatr Hematol Oncol. (2014) 36(2):96–8. 10.1097/MPH.0b013e31827e7f4c 23337552

[25] Bechard LJ Rothpletz-Puglia P Touger-Decker R Duggan C Mehta NM. Influence of obesity on clinical outcomes in hospitalized children: a systematic review. JAMA Pediatr. (2013) 167(5):476–82. 10.1001/jamapediatrics.2013.13 23478891 PMC4743026

[26] Krüger R Hanitsch LG Leistner R Schneider-Burrus S Hoppe PA Steinberg S Scabies, periorbital cellulitis and recurrent skin abscesses due to Panton-Valentine leukocidin-positive Staphylococcus aureus mimic Hyper IgE syndrome in an infant. Pediatr Infect Dis J. (2017) 36(12):e347–8. 10.1097/INF.0000000000001788 28938255

[27] Salleo E MacKay CI Cannon J King B Bowen AC. Cellulitis in children: a retrospective single centre study from Australia. BMJ Paediatr Open. (2021) 5(1):e001130. 10.1136/bmjpo-2021-001130 34337163 PMC8287612

[28] Cucka B Biglione B Xia J Tan AJ Chand S Rrapi R Complicated cellulitis is an independent predictor for increased length of stay in the neonatal intensive care unit. J Pediatr. (2023) 262:113581. 10.1016/j.jpeds.2023.113581 37353147

[29] Belinchón-Romero I Merino E Ramos-Rincón JM. Sex differences in clinical characteristics and outcomes in patients hospitalized with cellulitis in Spain (2016–2022). Int J Infect Dis. (2025) 154:107846. 10.1016/j.ijid.2025.107846 39961451

[30] Lizano-Díez I Naharro J Zsolt I. Indirect costs associated with skin infectious disease in children: a systematic review. BMC Health Serv Res. (2021) 21(1):1325. 10.1186/s12913-021-07189-3 34895206 PMC8665520

[31] Corrao G Rea F Mancia G. Evaluating sources of bias in observational studies. J Hypertens. (2021) 39(4):604–6. 10.1097/HJH.0000000000002725 33649281

